# A neurorobotic platform to test the influence of neuromodulatory signaling on anxious and curious behavior

**DOI:** 10.3389/fnbot.2013.00001

**Published:** 2013-02-05

**Authors:** Jeffrey L. Krichmar

**Affiliations:** ^1^Department of Cognitive Sciences, University of California, IrvineIrvine, CA, USA; ^2^Department of Computer Science, University of California, IrvineIrvine, CA, USA

**Keywords:** neuromodulation, anxiety, computer simulation, robotics, dopamine, serotonin, acetylcholine, norepinephrine

## Abstract

The vertebrate neuromodulatory systems are critical for appropriate value-laden responses to environmental challenges. Whereas changes in the overall level of dopamine (DA) have an effect on the organism's reward or curiosity-seeking behavior, changes in the level of serotonin (5-HT) can affect its level of anxiety or harm aversion. Moreover, top-down signals from frontal cortex can exert cognitive control on these neuromodulatory systems. The cholinergic (ACh) and noradrenergic (NE) systems affect the ability to filter out noise and irrelevant events. We introduce a neural network for action selection that is based on these principles of neuromodulatory systems. The algorithm tested the hypothesis that high levels of serotonin lead to withdrawn behavior by suppressing DA action and that high levels of DA or low levels of 5-HT lead to curious, exploratory behavior. Furthermore, the algorithm tested the idea that top-down signals from the frontal cortex to neuromodulatory areas are critical for an organism to cope with both stressful and novel events. The neural network was implemented on an autonomous robot and tested in an open-field paradigm. The open-field test is often used to test for models anxiety or exploratory behavior in the rodent and allows for qualitative comparisons with the neurorobot's behavior. The present neurorobotic experiments can lead to a better understanding of how neuromodulatory signaling affects the balance between anxious and curious behavior. Therefore, this experimental paradigm may also be informative in exploring a wide range of neurological diseases such as anxiety, autism, attention deficit disorders, and obsessive-compulsive disorders.

## Introduction

The vertebrate neuromodulatory systems are critical for appropriate value-laden responses to environmental challenges (Krichmar, 2008). Whereas changes in the overall level of dopamine (DA) have an effect on the organism's reward or curiosity-seeking behavior (Schultz et al., 1997; Berridge, 2004), changes in the level of serotonin (5-HT) can affect its level of anxiety or harm aversion (Millan, 2003; Cools et al., 2008). The cholinergic (ACh) and noradrenergic (NE) systems affect the ability to filter out noise and irrelevant events (Vankov et al., 1995; Bucci et al., 1998; Aston-Jones and Cohen, 2005; Yu and Dayan, 2005). These neuromodulatory systems have broad and extensive projections to the central nervous system causing shifts in behavior and learning.

The frontal cortex, which projects to all the neuromodulatory systems (Briand et al., 2007), may be carrying a level of cognitive control through modulating the neuromodulators. For example, the medial prefrontal cortex (mPFC) can control the stress response by its interaction with the raphe nucleus, the main source of 5-HT in the central nervous system (Jasinska et al., 2012), and the orbitofrontal cortex (OFC) may exert control on the DA reward system (Frank and Claus, 2006). Empirical evidence and theoretical modeling have suggested that the mPFC, the anterior cingulate cortex, and the OFC control decision-making in the face of reward-cost tradeoffs (Rudebeck et al., 2006; Rushworth et al., 2007; Chelian et al., 2012). That is, the OFC's interaction with the DA system is monitoring the expected reward of an action, and the mPFC's interaction with the 5-HT system is monitoring the expected cost of an action (Zaldivar et al., 2010; Asher et al., 2012).

Previously, a general-purpose algorithm, based on principles of the brain's neuromodulatory systems, was presented for action selection in robots (Krichmar, 2012). Rather than presenting a neurobiologically detailed model of how the nervous system achieves this function through neuromodulation [see for example (Cox and Krichmar, 2009)], a general-purpose, but minimal model of neuromodulatory function was developed, which could be applied to robot control. Similar to classic robot control algorithms, such as subsumption architecture (Brooks, 1991) and behavior-based schemas (Arkin, 1998), the algorithm automatically arbitrated between actions based on current sensory input. The algorithm demonstrated the ability to adapt to changes in the environment by: (1) increasing sensitivity to sensory inputs, (2) responding to unexpected or rare events, and (3) habituating or ignoring uninteresting events. The algorithm showed several important features for autonomous robot control in general, such as, fluid switching of behavior, gating in important sensory events, and separating signal from noise.

The present paper extends this algorithm in several key ways to make it more neurobiologically realistic, and more adaptable. First, a frontal cortex layer, which loosely corresponds to the OFC and mPFC and projects to the DA and 5-HT systems, respectively, is added to the model. This provides a degree of top-down control on the neuromodulatory systems that handle sensory events. Second, an inhibitory projection from the 5-HT system to the DA system was added based on evidence that these systems are somewhat in opposition (Tops et al., 2009; Boureau and Dayan, 2011). From a behavioral standpoint, the 5-HT system causes the organism to be withdrawn and risk-averse, and the DA system causes the organism to be invigorated and risk-taking. From the algorithm's standpoint, this allowed sensory events to be shared with the appropriate action taken based on the current levels of DA and 5-HT. Lastly, a variable was added to model the tonic levels of DA and 5-HT. The previous model only considered phasic neuromodulatory responses, which resulted in decisive action. The tonic levels in the present model can set the agent's behavioral context or state and make the agent more likely to select a particular set of actions.

The present algorithm tested the hypothesis that high levels of 5-HT lead to withdrawn behavior by suppressing DA action and that high levels of DA or low levels of 5-HT lead to curious, exploratory behavior. It has been suggested that serotonin opposes activating or invigorating neuromodulators such as dopamine (Tops et al., 2009). Specifically, projections from raphe serotonin cells to DA areas may oppose the action of DA and mediate avoidance of threats (Deakin, 2003). Furthermore, the algorithm tested the idea that top-down signals from the frontal cortex to neuromodulatory areas are critical for an organism to cope with both stressful and novel events. A recent review suggested that the mPFC inhibited the serotonergic raphe nucleus after handling a stressful event (Jasinska et al., 2012). This feedback loop prevented the raphe from being overly active after the stressor had been handled. The present algorithm further suggests that projections from the OFC to the dopaminergic ventral tegmental area (VTA) have a similar function when responding to a positive valence event.

The algorithm was implemented in a neural network that controlled the behavior of an autonomous robot and tested in the open-field paradigm. The open-field test is often used for animal models anxiety or exploratory behavior and allows for qualitative comparisons with the neurorobot's behavior (Heisler et al., 1998; Lacroix et al., 2000; Lipkind et al., 2004; Fonio et al., 2009).

## Methods

### Robot control

**Figure 1:**
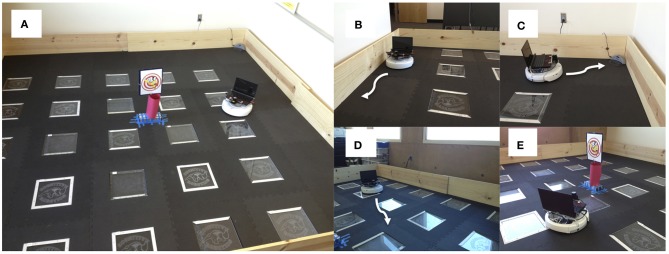
**Setup for neurorobotic experiments.** Experiments were run on an iRobot Create equipped with an URG-04-LX laser range finder (Hokuyo Automatic Co. LTD.) and a System 76 netbook running the Ubuntu Linux operating system for computation. **(A)** Environment was a 3.7 m^2^ arena enclosed with plywood. The picture in the middle was a novel object for the robot to explore. **(B)** Wall following behavior. Wall following was achieved using the Create's “Mouse” demo. **(C)** Find home behavior. Finding the docking station was achieved using the Create's “Cover and Dock” demo. **(D)** Open-field behavior. The robot moved toward open spaces in the environment based on laser range finder readings. **(E)** Explore object. The robot approached narrow objects based on laser range finder readings.

Experiments were run on an iRobot Create equipped with an URG-04-LX laser range finder (Hokuyo Automatic Co. LTD.) and a System 76 netbook running the Ubuntu Linux operating system for computation (see Figure 1). The Matlab Toolbox for iRobot Create (http://www.usna.edu/Users/weapsys/esposito/roomba.matlab/) was used to interface with the robot. The neural simulation and robot control algorithm for iRobot Create was written in Matlab (MathWorks) and can be downloaded at: http://www.socsci.uci.edu/~jkrichma/krichmar_frontiers2012_carl_roomba.m

Robot control was achieved through processing events and states. States were pre-canned behaviors and events were driven by sensory signals. An event could cause a switching of behavior states. The neural simulation, which is described below, arbitrated between incoming events and decided when to switch states. A simulation cycle, *t*, occurred approximately once per second, which was roughly the time needed to read CarlRoomba's sensors, update the neural simulation, and send a motor command to CarlRoomba. The main limitation for cycle duration was Matlab handling of I/O. Future versions of the software will be written in C/C++ to speed up I/O and shorten simulation cycles.

In the present experiments, the robot, which is called CarlRoomba, handled three events: (1) *Object Detected*. This event was triggered if the laser detected an object between 12 and 30 degrees wide and closer than one meter. (2) *Light detected*. This event was triggered if the average pixel brightness in the grayscale image was greater than 50%. The netbook's built-in camera was used to detect light levels. (3) *Bump detected*. This event was triggered by iRobot Create's bump sensors or if the laser detected an object closer than 20 cm.

CarlRoomba switched between four behavior states: (1) *Wall Follow* (Figure 1B). Wall following was achieved by calling the iRobot Create's mouse demo routine. This caused CarlRoomba to follow the wall to its right. (2) *Find Home* (Figure 1C). Find home was achieved by calling the iRobot Create's cover and dock demo routine. This caused CarlRoomba to move in a random pattern until it detected the Roomba docking station via an IR beam that had a range of roughly 500 cm. (3) *Open-Field* (Figure 1D). CarlRoomba would drive toward the most open area of the environment, as judged by the laser range finder. If a collision with an object was detected, CarlRoomba would rotate clockwise. (4). *Explore Object* (Figure 1E). CarlRoomba would move toward the object found by the laser. If a collision with an object was detected, CarlRoomba would rotate clockwise.

### Neural simulation

**Figure 2:**
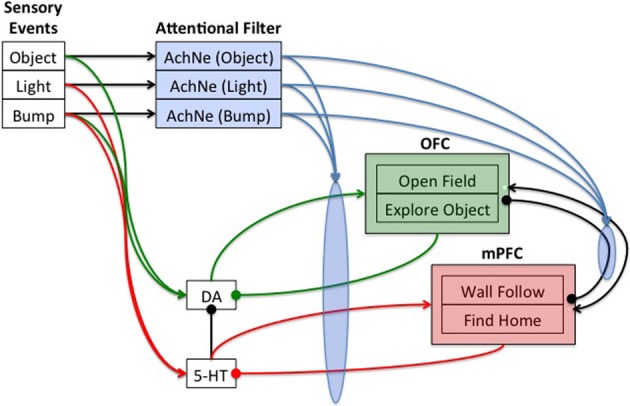
**Neural architecture to control robot behavior.** Sensory events were handled by three binary neurons. These neurons projected to the attentional filter neurons (AchNE) and the dopaminergic and serotonergic neurons (DA and 5-HT). The DA and 5-HT neurons projected to the OFC and mPFC neurons. The most active OFC or mPFC neuron dictated the robot's behavioral state. The AChNE neurons had a modulatory effect on the projection from the DA and 5-HT to OFC and mPFC (see blue ellipse and arrows). OFC and mPFC projected to 5-HT and DA neurons with inhibitory connections. Excitatory and inhibitory connections within and between OFC and mPFC neurons were all-to-all. See text for details.

Neuromodulatory systems receive sensory information and drive behavior by innervating downstream neural systems. The general framework of the present architecture is that sensory events can trigger neuromodulatory systems, which in turn drive behavior states (see Figure 2). Frontal areas (see OFC and mPFC in Figure 2) trigger action selection and exert cognitive control on the neuromodulatory areas (see DA and 5-HT in Figure 2) via inhibitory projections. The ACh and NE systems (see AChNE in Figure 2) act as an attentional filter allowing novel and unexpected events to gate through to the frontal cortex. Specifically, AChNE modulates connections from DA and 5-HT to cortical neurons and inhibitory connections between cortical neurons (see blue arrows and ellipses in Figure 2). It has been suggested that ACh and NE neuromodulation gates in sensory inputs and increases competition among frontal cortex neurons by up-regulating GABAergic currents, but not glutamatergic connections (Hasselmo and McGaughy, 2004; Aston-Jones and Cohen, 2005). Although the architecture given in Figure 2 is specific to the present problem space, the general framework could potentially be used to arbitrate any combination of sensory events and behavioral states.

In the present paper, the neural simulation consisted of three event neurons, each of which corresponded to one of the sensory events described above, four state neurons, each of which corresponded to one of the behavioral states described above, and neuromodulatory neurons. There was one DA neuron, one 5-HT, and three ACh/NE neurons, each of which corresponded to one of the sensory events described above. Figure 2 shows the architecture and connectivity of the network.

Initial simulations were carried out to set the weights and parameters given in the equations below. Weights were chosen such that the network demonstrated stable activity, and such that a phasic burst of neuromodulatory activity could efficiently drive action selection. Each OFC and mPFC neuron was connected to every other OFC and mPFC neuron with both excitatory (weight = +1.0) and inhibitory (weight = −1.0) connections. OFC neurons for OpenField and ExploreObject projected to the DA neuron with a weight equal to −1.0, and mPFC neurons for WallFollow and FindHome projected to the 5-HT neuron with a weight equal to −1.0. Neuromodulatory neurons selectively connected to OFC and mPFC neurons with weights set at 5, event neurons selectively connected to neuromodulatory neurons with weights set at 0.5, and event neurons connected to the corresponding ACh/NE neurons with weights set at 1.

In the present simulation, detecting an object with the laser signaled novelty or something potentially rewarding in the environment and worth taking a risk to investigate. Therefore, these events triggered dopaminergic neurons (Object→DA in Figure 2). A bright light signaled a potential danger, and thus triggered serotonergic neurons (Light→5-HT in Figure 2). A bump could signal either something interesting or noxious in the environment. Therefore, the bump event triggered both dopaminergic and serotonergic neurons (Bump→DA and Bump→5-HT in Figure 2). To model, serotonergic and dopaminergic opponency, 5-HT projected to DA with a weight set at −1.0.

Event neurons were binary and set to 1 when an event occurred and 0 otherwise. All other neurons were governed by the following activation function, which kept neural activity between 0 and 1:

(1) $$n(t)=\frac{1}{1+e^{-gI(t)}}$$

where *g* was the gain of the function and *I* was the input to the neuron. The initial weights, gains, and the baseline input, given in Equation 2, were set such that the range of synaptic input to the neuron would cover the full range of the sigmoid curve. Therefore, the gain was set to 2 for frontal cortex and neuromodulatory neurons, and 10 for ACh/NE neurons. Input to the neuron was based on pre-synaptic neural activity, *n*_*j*_(*t*), previous neural activity, *n*_*i*_(*t* − 1), and neuromodulation:

(2) $$I_{i}(t)=b+\sum_{j}n_{j}(t)w_{ji}(t)+pn_{i}(t-1)+{\text{tonic}}_{nm}(t)$$

where *b* was the baseline input set to −1.0 for DA and 5-HT, −0.5 for ACh/NE, and a random number that was drawn uniformly between negative one and zero for OFC and mPFC neurons. The baseline input was set such that the full range of the sigmoid curve (0 to 1 in Equation 1) was covered, and the random number value for *b*, which was drawn every time step for OFC and mPFC, added some stochasticity to cortical neural activity. *p* was the persistence set to 0.25 for frontal cortex, 0.5 for ACh/NE neurons, and zero for DA and 5-HT neurons. Synaptic input into neuromodulatory neurons had an additional term for tonic neuromodulation (tonic_*nm*_). For all other neurons, tonic_*nm*_ was set to zero.

In our previous model, the ACh and NE system was introduced as an attentional filter (Krichmar, 2012). When the ACh/NE system was impaired in the algorithm, the robot lost its ability to filter out noise and responded to any incoming sensory event. This attentional filter, which is shown pictorially in Figure 2 (see blue ellipse and arrows), was achieved by adding the following term to the synaptic input into OFC and mPFC neurons.

(3) $$I_{i}(t)=I_{i}(t)+\sum_{j}AChNE(t-1)n\_fctx_{j}(t-1)w\_inh_{ji}(t-1)+\sum_{k}AChNE(t-1)n\_nm_{k}(t-1)w\_nm_{ki}(t-1)$$

where AChNE is the sum of all neural activity in the ACh and NE areas, *n*_*fctx*_*j*_(*t*) is the activity from other frontal cortex neurons, *n*_*nm*_*k*_(*t*) is the neuromodulatory input into a frontal neuron, *w*_*inh*_*ji*_(*t*) is the weight of lateral inhibition from frontal cortex neuron *j* to frontal cortex neuron *i*, and *w*_*nm*_*ki*_(*t*) is the weight of the connection from neuromodulatory neuron *k* to frontal cortex neuron *i*.

AChNE neurons acted as an attentional filter for events by adjusting weights from event neurons to AChNE neurons through the following update rule:

(4) $$w_{ji}(t)=\{\begin{array}{l} p*wji(t-1)\text{ if }e_{j}=1\\ w_{ji}(t-1)+\frac{1-w_{ji}(t-1)}{\tau }\text{ otherwise} \end{array}$$

where *j* is the index of the event neuron, *i* is the index of the ACh/NE neuron, *p* is the amount of change in response to an event, and τ, which was set to 25, was a time constant that governed the rate at which weights returned to their original value. Weights from event neurons to ACh/NE neurons were depressing, meaning that each event caused the weight to decrease (*p* = 0.25).

Tonic activity in the DA and serotonergic neurons was modeled by having a facilitating response to sensory events gated in by the AChNE neurons:

(5) $${\text{tonic}}_{i}(t)=\{\begin{array}{l} p*{\text{tonic}}_{i}(t-1)\text{ if }AChNE_{j}>0.5\\ {\text{tonic}}_{i}(t-1)+\frac{1-{\text{tonic}}_{i}(t-1)}{\tau }\text{ otherwise} \end{array}$$

where *i* is the index of the neuromodulatory neuron, *j* is the index of the ACh/NE neuron, *p* is the amount of change in response to an event. The tonic levels rose every time there was a salient sensory event by setting *p* = 1.25. The time constant, τ, was related to neurotransmitter re-uptake, that is, how long a neuromodulator acted on its target neurons. For example, a larger value of τ meant that the re-uptake of a neuromodulator was slower and therefore the neuromodulator had a longer lasting effect. Initially, tonic_5HT_ was set to 2.0 and tonic_DA_ was set to 1.0, which caused CarlRoomba to have higher levels of 5-HT at the start of an experimental trial.

These rates and parameters were set based on the expected occurrence of events during a four-minute session of running CarlRoomba. For example, in the control condition, the parameters *p* and τ were chosen such that salient events would trigger a long lasting increase in tonic neuromodulation. Multiple events should cause a change in the neurorobot's contextual state (e.g., become withdrawn) and a long interval between events would result in the neurorobot settling into a neutral state. In other conditions, parameter τ was set to demonstrate how low and high levels of tonic neuromodulation, relative to the control condition, might affect behavior.

Action selection occurred after the neural activities and weight updates were calculated. The maximally active state neuron was chosen as the new behavioral state if it had activity greater than 0.67. This threshold was set such that new actions would be selected roughly 4–5 times per minute. If no state neuron was above this threshold, the previous behavioral state continued.

### Experimental paradigm

Experiments were run in an open-field arena, which was a 3.7 m^2^ region blocked off by plywood (see Figure 1A). A cardboard column and picture that was detectable by the laser was placed in the center of the arena. The Roomba docking station was placed in one corner of the arena. Experiments were run in the dark for 240 s. At approximately 120 s into an experiment, which allowed CarlRoomba to acclimate to the environment, the lights were turned on for 10 s and then turned off again. CarlRoomba always started the experiment in the corner of the arena where the docking station was located, and always faced the center of the arena. Each parameter setting was run 5 times on CarlRoomba, each with different random number generator seeds.

The experimental setup was designed to mimic a rodent open-field experiment and CarlRoomba's ability to handle a stressful event. When placed in a new environment, rodents typically stay near their nest (i.e., the docking station) or follow closely along the walls of an environment (Fonio et al., 2009). As they become more comfortable in the environment, they will venture out into the open area of the arena or explore a novel object placed in the arena. This paradigm is often used to test animal models of anxiety (Simon et al., 1994; Heisler et al., 1998; Lipkind et al., 2004). The present experiments were designed to test how dopaminergic and serotonergic neuromodulation influence the ability to cope with a stressful event. In Fonio's experimental paradigm, the moving of a mouse to a novel environment is presumably a stressful event. However, this prior context would be difficult to mimic with the neurorobot CarlRoomba. Therefore, a light flash was used to mimic a stressful event in the open-field test, since rodents typically prefer the dark.

## Results

### Cognitive control of interesting and stressful events

**Figure 3:**
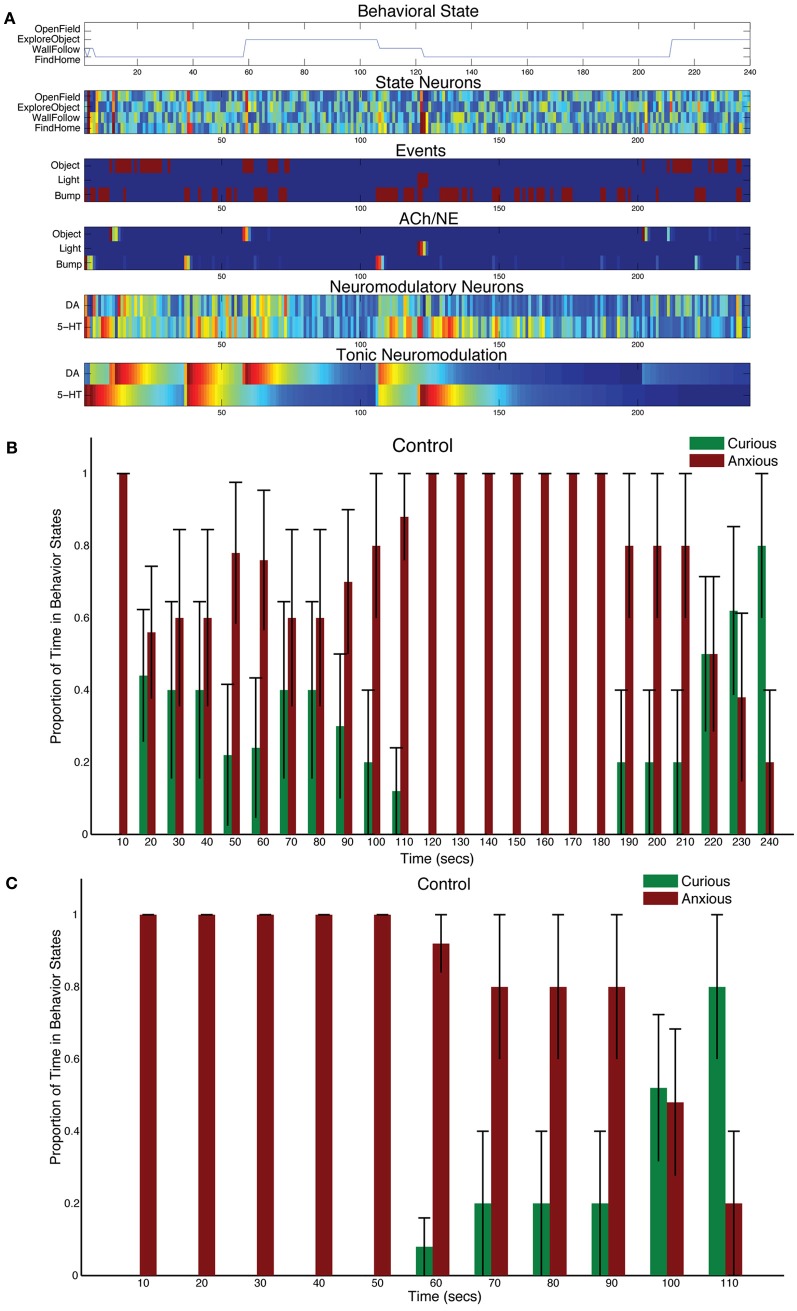
**Behavioral and neural responses in the intact model.** The time constants τ_DA_ and τ_5−HT_ were both set at 50. **(A)** Behavioral and neural responses in a representative trial. The x-axis for all charts shows the time of the trial in seconds. The chart labeled “Behavioral State” denotes the state of the robot at a given time. The charts labeled “State Neurons,” “Events,” “ACh/NE,” and “Neuromodulatory Neurons” show the neural activity over the trial, where dark blue equates to no activity and bright red equates to maximal activity. Note that Event neurons were binary. The chart labeled “Tonic Neuromodulation” denotes the level of tonic activation contributing to DA and 5-HT neurons. **(B)** The proportion of Curious (ExploreObject and OpenField) and Anxious (FindHome and WallFollow) behavior averaged over 5 trials. The error bars denote the standard error. The histogram binned the behavior in 10 s windows. **(C)** Similar to **(B)** except the behaviors were time-locked to the Light event.

CarlRoomba responded appropriately to sensory events in its environment. Novel objects resulted in it exploring the environment, stressful events, such as bright lighting caused it to seek safety. Figure 3A shows a representative trial from a CarlRoomba where there were balanced tonic levels of neuromodulation (τ_DA_ = τ_5HT_ = 50 in Equation 5). In Figure 3A and subsequent representative trial figures, the x-axis denotes time in seconds from the start of the trial until the end, which was approximately 240 s. The upper chart shows CarlRoomba's behavioral state over the course of the trial. The second through fifth charts show the neural activity of the State, Event, ACh/NE, and Neurmodulatory neurons, respectively, over the course of a trial where dark blue signifies no activity and bright red signifies maximal activity. The bottom chart denotes the level of tonic neuromodulation (see Equation 5). Note how initially when CarlRoomba was unfamiliar with the environment, serotonergic activity dominated, resulting in anxious behavior, such as WallFollow and FindHome actions. However, as CarlRoomba became more familiar and comfortable in its environment (approximately 60 s into the trial), DA levels were higher and there was more curious or exploratory behavior. Note that the AChNE neurons only gated through interesting and rare events. This was achieved through AChNE modulation of projections from neuromodulatory neurons to OFC and mPFC and through AChNE modulation of intrinsic inhibitory projections between frontal cortex neurons (see Equations 3 and 4 and Figure 2). For example, constant bump events were habituated (compare Bump event neuron activity with Bump AChNE activity in Figure 3A). At approximately 120 s into the trial, there was an unexpected Light event, which resulted in a phasic 5-HT response and a longer tonic increase in 5-HT (see Equations 2 and 5). This caused CarlRoomba to respond with withdrawn or anxious behavior until approximately 210 s into the trial when a pair of object events triggered exploration of the center of the environment (see Figure 3A). Specifically, tonic levels of 5-HT had decayed and the object events caused an increase in DA levels triggering a change in behavioral state.

Figure 3B shows the proportion of curious behavior (OpenField and ExploreObject) and anxious behavior (FindHome and WallFollow) for five experimental trials. In Figure 3B and subsequent figures summarizing five trials, histograms were calculated with 10 s bins over the course of the trial. Each bar was the average proportion of time spent in either curious (green bars) or anxious behavior (red bars) in a 10 s period of the trial. The error bar denoted the standard error. Note that on different trials, the timing of the light event varied (as early as 118 s and as late as 130 s). Thus, the increase in “Anxious” behavior at 110 s (see Figure 3B) is not due to a prediction of the stressful event, but rather trial variation. Because the initial state of CarlRoomba is not necessarily anxious or curious, and CarlRoomba pointed toward the center of the arena at the start of every trial, it is hard to quantify CarlRoomba's behavior over the first half of each trial. However, CarlRoomba's initial behavior appeared to be anxious, and then more curious as it became more familiar with the environment.

To resolve potential issues with comparing across conditions that result from trial and initial state variation, Figure 3C and subsequent population figures shows the behavior time-locked to the light event. The light event, which occurred at approximately the halfway point in the trial, was introduced to cause a stress response in CarlRoomba (see Figure 3). The ability of CarlRoomba to handle this stressful event was compared across all conditions. After the light event, the neurorobots' behavior rapidly switched to anxious behavior until roughly 200 s when it became curious again (see Figure 3C). Variation occurred due to different times of the light event, and random variations in other sensory events.

The neurorobots' behavior after a stressful event was qualitatively similar to a rodent's behavior when placed in a novel environment. For example, in Fonio et al.'s experiments (Fonio et al., 2009), mice progressed from staying near a nest (1–4 in their developmental sequence in Fonio et al., 2009, Figure 1), making circuits along the border of the environment (5–9 in Fonio et al., 2009, Figure 1), and then crossing the center of the environment (10–11 in Fonio et al., 2009, Figure 1). All their mice followed this behavioral pattern. In a similar way, CarlRoomba followed this pattern. In all five trials for the first 50 s following the light flash, CarlRoomba stayed near its docking station and the walls of the arena. By 100 s after the light flash, CarlRoomba spent over half its time either crossing the center of the environment or investigating a novel object in the center of the environment. These control experiments show that when CarlRoomba has an intact nervous system, it is able to respond appropriately to a stressor, and then resume exploratory behavior when the stressor has passed.

### Serotonin and the ability to cope with stressful events

It has been suggested that degradation of serotonin re-uptake can have detrimental effects on the ability to cope with stressors (Jasinska et al., 2012). To mechanistically test this notion, the time constant for tonic serotonin was increased (τ_DA_ = 50, and τ_5HT_ = 150 in Equation 5). This had the effect of serotonin staying in the system longer after a stressful event.

**Figure 4:**
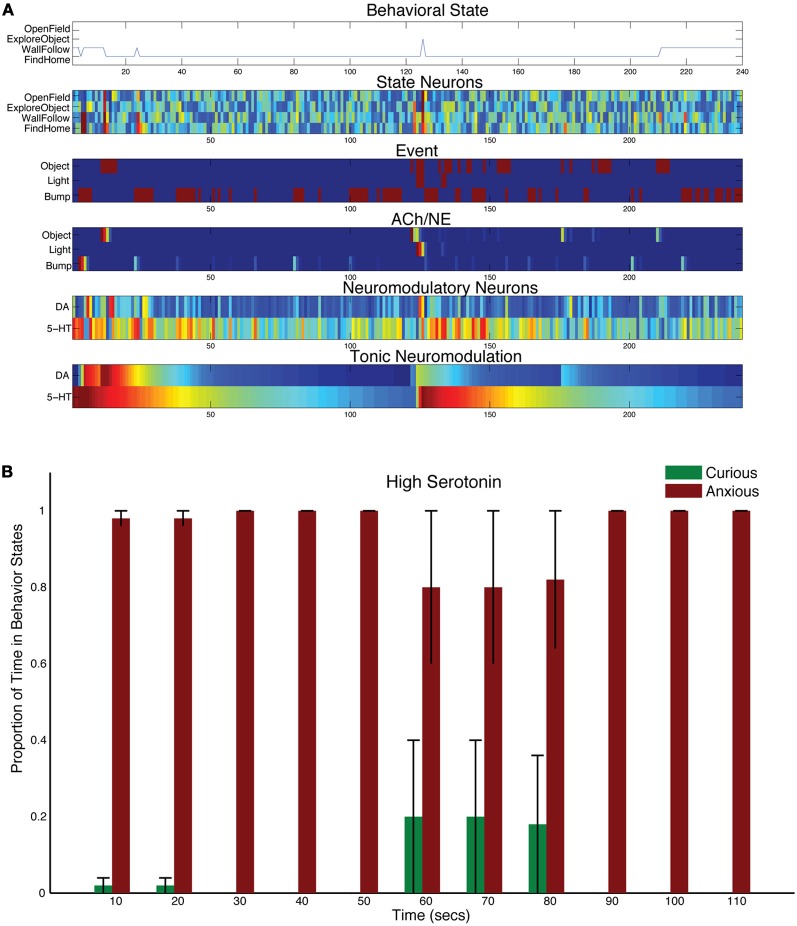
**Behavioral and neural responses with high serotonin levels.** The time constant τ_DA_ was set to 50 and the time constant τ_5−HT_ was set to 150. **(A)** Behavioral and neural responses in a representative trial. Axes, labels, and color are the same as in Figure 3A. **(B)** The proportion of Curious (ExploreObject and OpenField) and Anxious (FindHome and WallFollow) behavior averaged over 5 trials. Axes, labels, and time locking is the same as in Figure 3C.

A stressful event, such as a bright light, still caused CarlRoomba to select anxious behaviors, but the increase in serotonin levels resulted in CarlRoomba never breaking out of this stressful behavior. Figure 4A shows a representative trial where τ_5HT_ was longer. Compared to Figure 3A, serotonin levels remain high and the resulting behavior is almost entirely wall following and finding home. Figure 4B shows the population behavior of five trials time locked to the light event. As in the control case, there is a strong response to the light. However, unlike the control behavior shown in Figure 3C, CarlRoomba with high serotonin levels never recovers from this stressful event, and demonstrates anxious behavior throughout the remainder of the trial. These results are qualitatively similar to that shown by Heisler and colleagues where genetically mice that were lacking in 5HT1A receptors spent less time in the center of the open-field arena (Heisler et al., 1998). 5-HT1A receptors located on serotonergic neurons act as autoreceptors and suppress serotonergic neuronal activity. Therefore, mice lacking in 5HT1A would have increased levels of serotonin in the nervous system. In the open-field test, these mice showed reduced time in the center of the arena, and were less likely to approach a novel object.

**Figure 5:**
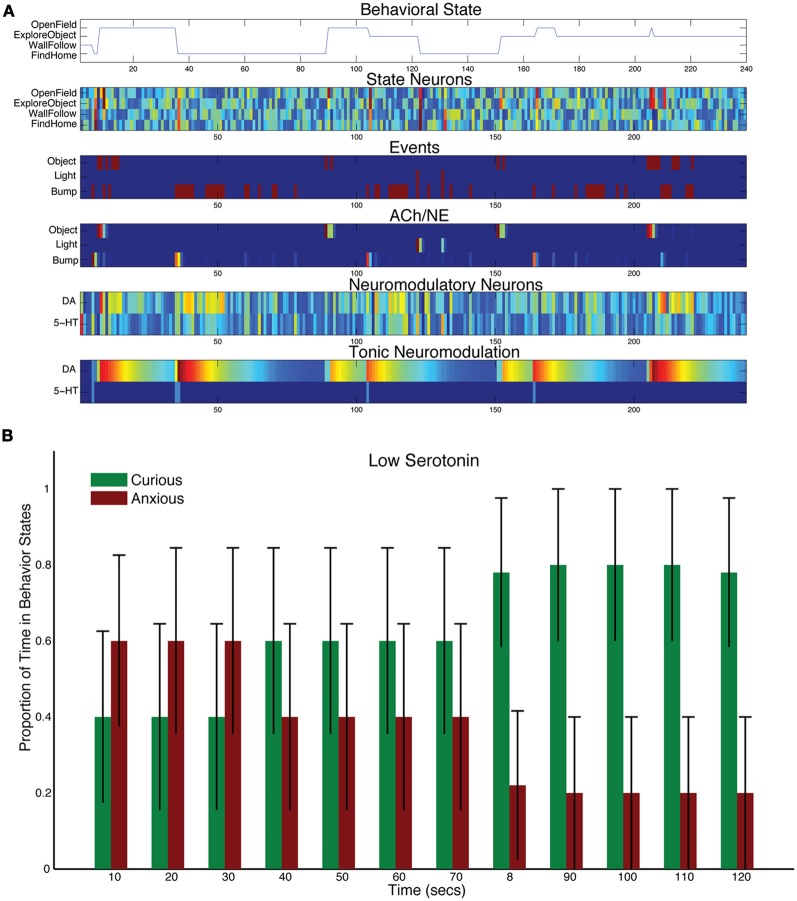
**Behavioral and neural responses with low serotonin levels.** The time constant τ_DA_ was set to 50 and the time constant τ_5−HT_ was set to 1. **(A)** Behavioral and neural responses in a representative trial. Axes, labels, and color are the same as in Figure 3A. **(B)** The proportion of Curious (ExploreObject and OpenField) and Anxious (FindHome and WallFollow) behavior averaged over 5 trials. Axes, labels, and time locking is the same as in Figure 3C.

To test how lowering levels of serotonin affect behavior, the time constant for tonic serotonin was lowered with respect to control levels (τ_DA_ = 50, and τ_5HT_ = 1 in Equation 5). This drastically reduced the tonic levels of serotonin in the model, but the serotonergic system still responded phasically to sensory events (see Figure 5A). For example, there was a serotonergic response to the light event at 120 s into the trial. However, the object sensory event at 150 s and the bump event at 160 s resulted in CarlRoomba taking exploratory behavior. Figure 5B shows the population behavior of five trials time locked to the light event. There is still some response to the light with anxious behavior, but CarlRoomba quickly switches to more curiosity seeking behavior, much more so than in the control experiments (compare Figure 3C with Figure 5B), by moving to the open part of the arena and exploring the object in the center.

Lowering serotonin levels through Acute Tryptophan Depletion (ATD) has been shown to reduce harm aversion and increase risk taking in humans (Crockett et al., 2008; Robinson et al., 2010). This is qualitatively similar to CarlRoomba's increased tendency to explore after a stressful event. Interestingly, ATD increased anxious behavior in the open-field test with rats (Blokland et al., 2002). In their discussion, they state that ATD only moderately lowers serotonin levels in rats (40%), but has a stronger effect in humans (80–90%). This may explain the difference between CarlRoomba's behavior and Blokland and colleagues' experiments. Future experiments with only a moderate change to τ_5HT_ may resolve this difference.

### Dopamine and risk taking

**Figure 6:**
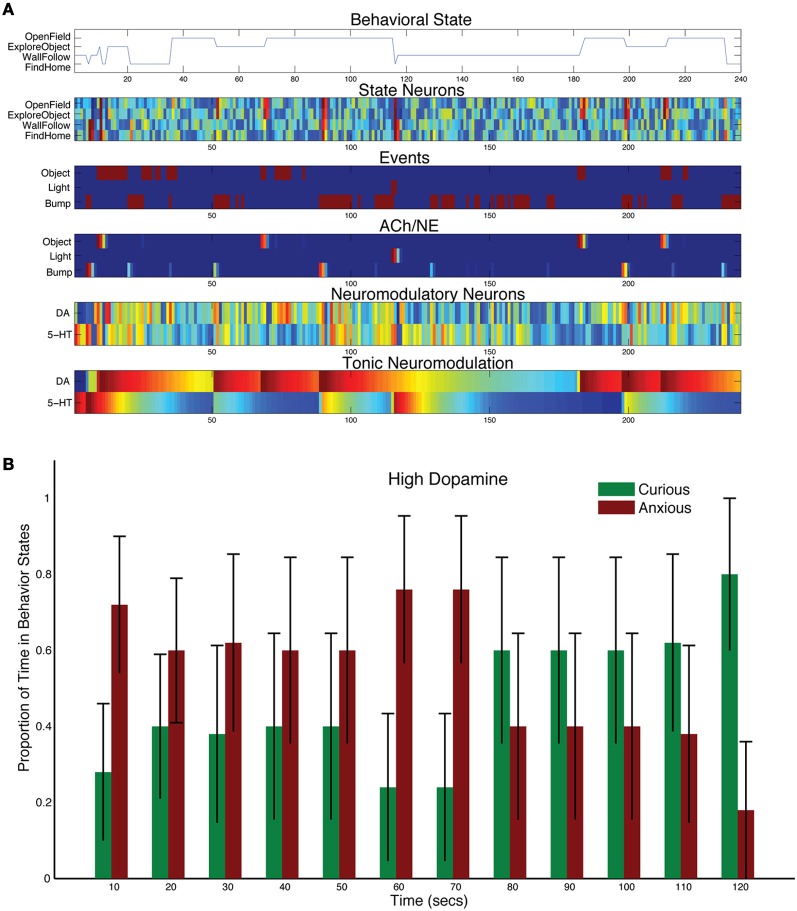
**Behavioral and neural responses with high dopamine levels.** The time constant τ_DA_ was set to 150 and the time constant τ_5−HT_ was set to 50. **(A)** Behavioral and neural responses in a representative trial. Axes, labels, and color are the same as in Figure 3A. **(B)** The proportion of Curious (ExploreObject and OpenField) and Anxious (FindHome and WallFollow) behavior averaged over 5 trials. Axes, labels, and time locking is the same as in Figure 3C.

Increasing the levels of DA by adjusting the tonic time constant (τ_DA_ = 150, and τ_5HT_ = 50 in Equation 5), resulted in more curiosity and risk taking, but did not abolish the stress response (see Figure 6B). For example, in the representative trial shown in Figure 6A, the light event did cause a strong increase in 5-HT activity, which in turn inhibited DA activity. However, the next sensory events, which were gated through by the AChNE attentional filter at approximately 180, 200, and 220 s, resulted in strong DA activation and curiosity seeking behavior. The population data reflected this interplay between the DA and 5-HT system. CarlRoomba responded to the stressful event, but was much more curious than controls. In effect, CarlRoomba was taking more risks by venturing into the middle of the environment during or right after the stressful light event. Similarly, cocaine, which increases levels of DA in the nervous system, has been shown to increase activity in the open-field test with rats, as well as increase the exploration of novel objects (Carey et al., 2008).

**Figure 7:**
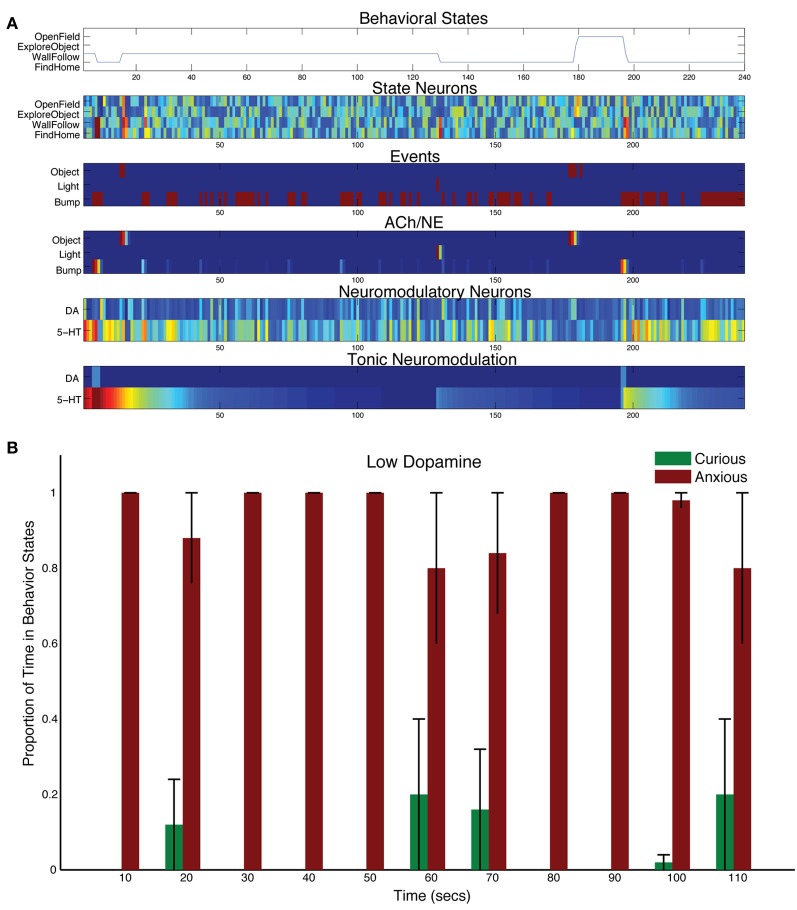
**Behavioral and neural responses with low dopamine levels.** The time constant τ_DA_ was set to 1 and the time constant τ_5−HT_ was set to 50. **(A)** Behavioral and neural responses in a representative trial. Axes, labels, and color are the same as in Figure 3A. **(B)** The proportion of Curious (ExploreObject and OpenField) and Anxious (FindHome and WallFollow) behavior averaged over 5 trials. Axes, labels, and time locking is the same as in Figure 3C.

Decreasing the levels of DA by adjusting the tonic time constant (τ_DA_ = 1, and τ_5HT_ = 50 in Equation 5) resulted in less curiosity, and more withdrawn behavior (see Figure 7). Object events did sometimes results in curious behavior (see 180 s into the trial shown in Figure 7A). But, in general, without much DA in the system, the 5-HT system dominated action selection leading to anxious behavior, such as following walls and searching for its home (i.e., docking station). For example, the bump event at 200 s into the trial in Figure 7A, triggered an anxious FindHome response by CarlRoomba. Overall, CarlRoomba's behavior was considerably more anxious when comparing the low DA condition (Figure 7B) to the control condition (Figure 3C).

### Frontal cortex and cognitive control

The OFC and mPFC areas of the model exert cognitive control on CarlRoomba's behavior by inhibiting the DA and 5-HT systems, respectively (see Figure 2). Activity in these areas initiated behavior selection, but also inhibited the neuromodulatory systems. This inhibition kept the appropriate neuromodulatory system in check and exerted cognitive control by signaling to the neuromodulatory system that the sensory event had been handled.

**Figure 8:**
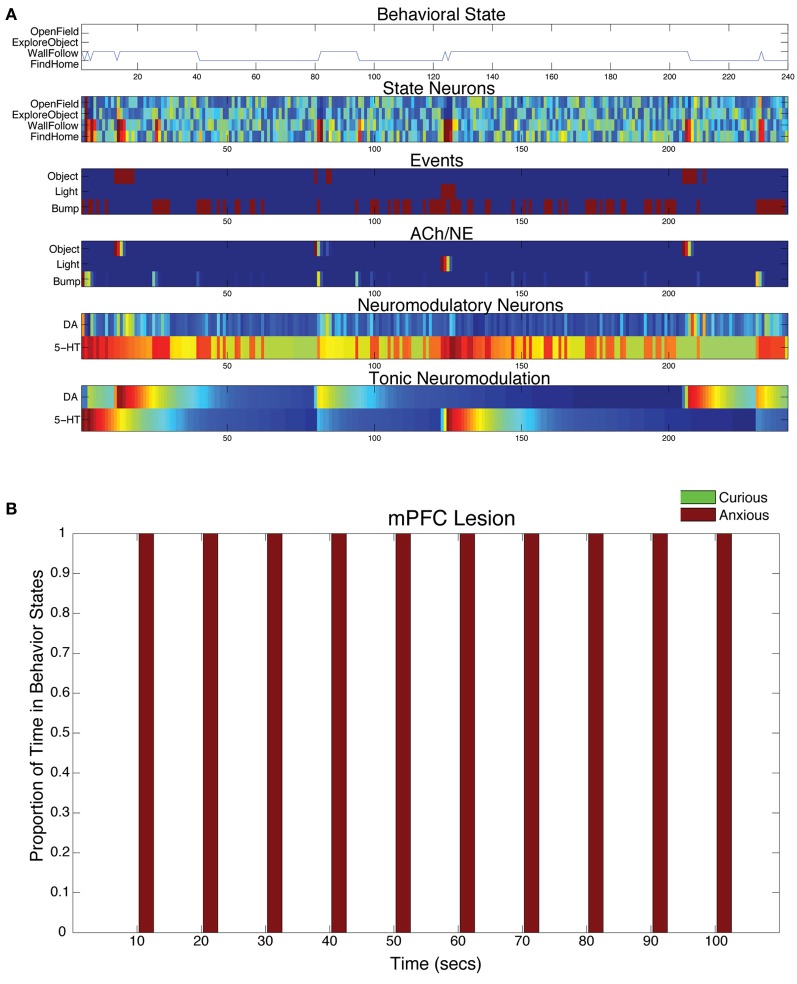
**Behavioral and neural responses with lesion to projection from mPFC to 5-HT.** The time constants τ_DA_ and τ_5−HT_ were both set at 50. **(A)** Behavioral and neural responses in a representative trial. Axes, labels, and color are the same as in Figure 3A. **(B)** The proportion of Curious (ExploreObject and OpenField) and Anxious (FindHome and WallFollow) behavior averaged over 5 trials. Axes, labels, and time locking is the same as in Figure 3C.

When the projections from mPFC to 5-HT were lesioned in the model, the serotonergic system was overactive and CarlRoomba acted anxious almost entirely (see Figure 8A). In all mPFC lesion cases, the light response triggered anxious behavior that persisted throughout the remainder of the trial (see Figure 8B).

**Figure 9:**
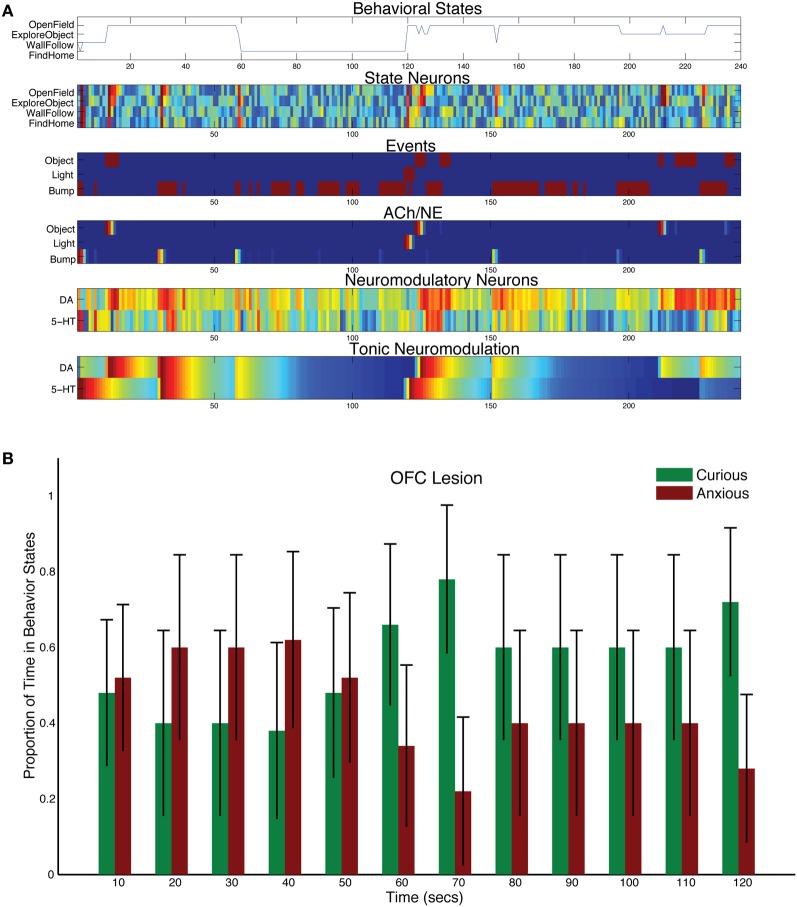
**Behavioral and neural responses with lesion to projection from OFC to DA.** The time constants τ_DA_ and τ_5−HT_ were both set at 50. **(A)** Behavioral and neural responses in a representative trial. Axes, labels, and color are the same as in Figure 3A. **(B)** The proportion of Curious (ExploreObject and OpenField) and Anxious (FindHome and WallFollow) behavior averaged over 5 trials. Axes, labels, and time locking is the same as in Figure 3C.

When the projections from OFC to DA were lesioned in the model, DA levels dominated and more exploratory behavior was observed (see Figure 9). Although CarlRoomba showed more curious behavior, anxious behavior was not abolished (compare Figure 8B with Figure 9B). The asymmetry between these lesion experiments may be due to the opponency between the serotonergic and DA systems. The serotonergic system, through its inhibition of the DA system, can still trigger anxious behavior in response to a stressful event and may keep DA levels in check.

## Discussion

The main purposes of the present neurorobotic study were to demonstrate that (1) high levels of serotonin lead to withdrawn behavior, and that (2) top-down signals from the frontal cortex to neuromodulatory areas are critical for coping with both stressful and novel events. Firstly, it has been suggested that serotonin opposes activating or invigorating neuromodulators such as dopamine (Tops et al., 2009). When the simulated nervous system was intact, the neurorobot appropriately responded to a stressful event with an increase in 5-HT activity. This led to withdrawn behavior by activating the mPFC and suppressing DA activity. Secondly, a recent review suggested that the mPFC inhibited the serotonergic raphe nucleus after handling a stressful event (Jasinska et al., 2012). In the present model, this feedback loop prevented the raphe from being overly active after the stressor had been handled. Over time, this allowed the DA system to become active leading to exploratory behavior. The present algorithm further suggested that projections from the OFC to the DA function have a similar function when responding to positive novel events. Lastly, the introduction of the attentional filter in the ACh and NE systems allowed the neurorobot to respond to novel events and habituate to irrelevant events. As was shown in Krichmar (2012), when the ACh/NE system was compromised, the neurorobot was distracted by irrelevant events and switched behaviors constantly.

The behavior of the robot was similar to that observed in rodents under similar conditions. Specifically, the neurorobot, CarlRoomba, and the rodent are initially anxious or wary, resulting in staying near their nest or the walls of the arena (Fonio et al., 2009). After becoming familiar with the environment, both the rodent and CarlRoomba made forays into the middle of the arena. Figure 3 summarizes this behavior in the neurorobot. Because CarlRoomba started each trial pointed directly at the object in the middle of the environment, there was some selection of OpenField and ExploreObject behaviors early on. In Fonio's experimental paradigm, the moving of a mouse to a novel environment is presumably a stressful event. However, this prior context would be difficult to mimic with the CarlRoomba. Therefore, a light flash was used to mimic a stressful event. In this case, CarlRoomba's behavior was qualitatively similar to the rodent. CarlRoomba tended to stay near its docking station or the walls of the arena. By 100 s after the light flash (see Figure 3C), CarlRoomba spent over half its time either crossing the center of the environment or investigating a novel object in the center of the environment.

Opponency between the serotonergic system and the DA system has been proposed behaviorally and in theoretical models (Daw et al., 2002; Tops et al., 2009). However, whether the anatomy supports uni-directional or bi-directional inhibition is an open issue (Boureau and Dayan, 2011). But there is evidence that projections from raphe serotonin cells to DA areas oppose the action of DA and mediate avoidance of threats (Deakin, 2003). Therefore, opponency in the present neurorobotic framework was modeled by inhibition from the raphe nucleus to the ventral tegmental area (shown as 5-HT→DA in Figure 2). There were also practical reasons for this projection. First, there was a need to arbitrate between sensory events that might trigger both DA and 5-HT, such as a bump event. Second, by having 5-HT inhibit DA, a bump event would cause anxious behavior early in a trial (Fonio et al., 2009) and after a stressor (Jasinska et al., 2012). This matches behavioral data and suggests that the serotonergic system may be actively opposing the dopaminergic system, and that dopaminergic system exerts its influence if serotonin levels are sufficiently low. Lastly, it may be advantageous, from a robot control perspective, to be initially conservative, but transition from conservative to riskier action over time if environmental conditions warrant such action.

### Serotonin and risk-averse behavior

The serotonergic system is involved in the control of anxious states (Millan, 2003). For instance, a variation of an upstream promoter region of the serotonin transporter gene (5-HTTLPR) has been shown to influence both behavioral measures of social anxiety and amygdala response to social threats in humans (Hariri et al., 2002; Caspi et al., 2003, 2010). Lowering serotonin levels, through a dietary manipulation called ATD, has been shown to decrease cooperation and lower harm-aversion (Wood et al., 2006; Crockett et al., 2008). Moreover, manipulations of 5-HT receptor genes have an impact on stress and anxiety in mice (Heisler et al., 1998; Weisstaub et al., 2006; Holmes, 2008).

These serotonin-dependent traits and responses were shown in the present robot experiments. Increasing serotonin levels by lengthening the time constant for tonic 5-HT had a similar effect to the short allele variant of 5-HTTLPR. The robot showed stronger and long-lasting responses to a stressful event, that is, a bright light (see Figure 4). Indeed, these open-field responses are in agreement with mouse behavior, where manipulations to 5-HT1A and 5-HT2A receptors resulted in elevated anxiety in the open-field test as measured by center locomotion, overall distance traveled, rearing, and response to a novel object (Heisler et al., 1998; Weisstaub et al., 2006).

Similar to the decrease in harm aversion shown due to ATD (Wood et al., 2006; Crockett et al., 2008), decreasing serotonin levels in the model, through shortening the 5-HT time constant, had the effect of making the robot more risk taking (see Figure 5). The robot made more forays into the center of the environment, and more explorations of the object in the center of the environment.

### Dopamine and risk-taking behavior

The DA system has been implicated in the prediction of rewards and incentive salience or “wanting” (Schultz et al., 1997; Berridge, 2004), as well as novelty-seeking (Redgrave and Gurney, 2006; Bromberg-Martin et al., 2010). Variations in the DA system have been shown to affect risk-taking during gambling, the ability to filter out noise, and cognitive flexibility (Winterer and Weinberger, 2004; Roussos et al., 2008). A blockade of DA resulted in rats not making an extra effort of climbing over a barricade to get a high reward (Denk et al., 2005). This might be interpreted as low DA levels lead to less risk taking for potential rewards. Similarly, a human study has shown that individuals with a COMT polymorphism, which lowered levels of DA in the prefrontal cortex, tended to take fewer risks in a gambling task (Roussos et al., 2008). Moreover, individuals with this polymorphism persisted in accordance with prior instructions despite evidence that the rules had changed (Doll et al., 2011). Genetic variation in the DA system also has an effect on impulsivity. Polymorphisms in DA-related genes, including variable number tandem repeat (VNTR) polymorphisms in DRD4 and DAT1, have been associated with poor “action restraint” and “action cancellation” (Congdon et al., 2008; Munafo et al., 2008).

These DA-dependent behaviors and responses were observed in the robot's behavior and simulated nervous system. Similar to the Denk and Roussos findings, lowering tonic levels of DA led to a lack of risk-taking and more withdrawn behavior (Denk et al., 2005; Roussos et al., 2008). This was mainly due to the serotonergic system dominating and driving harm aversive behaviors, such as finding home or wall following (see Figure 7). It also led to behavior that could be regarded as impulsive since CarlRoomba perseverated with these behaviors. However, when the DA levels were elevated, the robot tended toward curious behavior (see Figure 6). It is interesting that in this condition, compared to others, the change in behavior is not as dramatic. It makes the prediction that the “anxious” behavior system (i.e., mPFC←→5-HT) may keep the “curiousity-seeking” behavior system (i.e., OFC←→DA) somewhat in check.

### Frontal cortex and cognitive control

Recent experiments suggest that the reward and cost of actions are also partially represented in OFC and mPFC, respectively. In general, OFC appears to be involved in decision-making and planning with respect to rewards and preferences, and the mPFC appears to be involved in decision-making and planning having to do with effort, cost, and social valuation (Rushworth et al., 2007). Rudebeck et al., for example, trained rats to choose maze arms that yielded more food pellets either after a delay or after scaling a barrier (Rudebeck et al., 2006). When the OFC was lesioned, the rat was more likely to choose the lower (immediate) reward than the higher (deferred) reward. However, mPFC lesions, specifically the anterior cingulate cortex, caused rats to more often pick lower (less effortful) rewards than higher (more effortful) rewards. Moreover, unit recordings in the rat anterior cingulate cortex have shown that many of these neurons respond to effort during goal-directed actions (Cowen et al., 2012).

In the model, when CarlRoomba responded to a stressful event (e.g., bright light), there was first a phasic response in the 5-HT system, causing activity in the appropriate mPFC state neurons, resulting in the selection of a stress reducing behavior, and then the mPFC inhibited the 5-HT system, since it had dealt with the stressor. However, lesioning the connections from mPFC to the 5-HT system had a dramatic effect on behavior; anxious behavior completely dominated because cognitive control of the serotonergic systems was absent. CarlRoomba became withdrawn since the cognitive control of the serotonergic system was removed (see Figure 8).

Evidence suggests that mPFC mediates the cognitive control of stress by regulating the raphe nucleus (i.e., serotonergic system) (Maier and Watkins, 2010). In a study where rats were subjected to tailshocks, inactivation of the mPFC resulted in the elimination of the ability to control the stressor through regulation of raphe nucleus serotonin levels (Amat et al., 2005). Interestingly, Lacroix and colleagues found that lesions of the mPFC did not increase anxiety in rats during unconditioned fear paradigms, such as the open-field test, but increased anxiety during conditioning paradigms (Lacroix et al., 2000). The present model does not have the type of learning to support conditioning. Future models of CarlRoomba may need to investigate this dissociation with the addition of biologically plausible learning rules.

In a similar fashion to the model of mPFC's control of stress, CarlRoomba's OFC exerted control on incentive salience or reward-seeking. When CarlRoomba responded to a potentially interesting event, such as an object or a bump, there was first a phasic response in the DA system, causing activity in OFC state neurons, resulting in the choice of a reward-seeking behavior (e.g., OpenField or ExploreObject) and then the OFC inhibited the DA system, since it had responded to the event of interest. However, when the OFC was lesioned, the robot perseverated in its curious behavior (see Figure 9). In about 50% of the trials, CarlRoomba did not respond to the stressful light event and continued with its “Curious” behavior.

It has been suggested that the OFC is crucial for adaptation when reward values or contextual cues change (Rolls, 2004), and that the OFC is important for developing stimulus to reward associations, prediction, and expectancies (Schoenbaum et al., 2009). A recent rodent study showed that, depending on the conditions, the OFC is important for both of these roles (Riceberg and Shapiro, 2012). OFC lesions impaired reversal learning when the reversals occurred at low frequencies. However, when the contingencies changed at a high frequency, OFC lesions rats followed a Lose-Shift strategy. The authors suggest that OFC is computing reward expectancies based on reward history. Although CarlRoomba does not contain the learning machinery to calculate reward expectancies, it does show perseverative behavior when from the OFC to the DA system are lesioned. The OFC lesioned CarlRoomba also showed a lack of ability to assess the potential rewards for a given event (i.e., all events became highly rewarding). It will be of interest to add predictive reward learning (e.g., TD learning) to the model and test the system in a reversal learning task.

### Related work

While there have been many models of action selection, the present work addresses how principles of neuromodulation and frontal cortex control could control autonomous robot behavior. It should be noted that other neural systems support action selection and behavioral switching. For example, the basal ganglia and its interaction with thalamocortical loops have been proposed as an action selection system (Prescott et al., 2006). This model, which was tested on a neurorobot, demonstrated behavioral switching in an open environment during a foraging task where the robot switched between wall-seeking, wall-following, approaching and placing objects. Similar to the present model, this basal ganglia model was able to choose between multiple, conflicting choices based on its context and motivation.

The present model was specifically designed to test how the opponency between the serotonergic and dopaminergic system, combined with top-down control from frontal cortex, could replicate rodent behavior. Moreover, it was able to show how altering the balance between these systems could influence anxious and exploratory behavior. These results can be compared to rodent studies under similar condition as described above (Heisler et al., 1998; Lacroix et al., 2000; Blokland et al., 2002; Lipkind et al., 2004; Bouwknecht et al., 2007). Future experiments may further delineate the role of these neuromodulators in balancing exploratory and anxious behavior. Moreover, the present neurorobotic experiments tests the feasibility of the architecture proposed by Jasinska and colleagues, where there is interaction between the mPFC and the raphe nucleus, for handling stressful events (Jasinska et al., 2012). CarlRoomba's neural architecture further suggests that there is a similar architecture between the OFC and DA system for handling positive valence stimuli.

Theoretical models have been proposed on neuromodulation, but they typically have not considered all of the neuromodulatory systems and their interactions with cortical and subcortical areas. The phasic response of the DA system has been proposed to signal temporal difference error (Schultz et al., 1997). Following this idea, the phasic response of DA has been modeled to shape behavior and action selection with reinforcement learning (Krichmar and Edelman, 2002; Sporns and Alexander, 2002; Arleo et al., 2004; Iida et al., 2004; Doya and Uchibe, 2005; Stone et al., 2005; Guenter et al., 2007; Nakamura et al., 2007).

Several neurorobot and computational neuroscience studies have investigated the interaction between multiple neuromodulatory systems. Our previous model took into consideration the phasic aspects of dopaminergic and serotonergic neuromodulation (Cox and Krichmar, 2009). This model postulated, similar to a model of noradrenergic neuromodulation (Aston-Jones and Cohen, 2005), that phasic neuromodulation causes an organism to be more decisive, whereas a lack of phasic response would result in more arbitrary action selection. A recent neurorobot study combined DA reinforcement learning with an exploration parameter related to the noradrenergic system (Khamassi et al., 2011). These simulated neuromodulatory systems interacted with an anterior cingulate cortex and prefrontal cortex. On two different robot platforms, they demonstrated that their model could deal with both expected and unexpected uncertainties in the real world. Our group has recently investigated the possible role of multiple neuromodulators in a resource allocation task (Chelian et al., 2012), and reversal learning on an autonomous robot (Oros and Krichmar, 2012).

However, few researchers have developed a model that includes the ACh, DA, NE, and 5-HT systems simultaneously. One exception was a theory proposed by Kenji Doya (Doya, 2002, 2008). In this theory, Doya subscribed a different functional role for each neuromodulatory system on different parameters of the temporal difference learning rule. Although this idea has not been implemented in a behaving robot, their group is actively exploring elements of this theory experimentally (Tanaka et al., 2007; Schweighofer et al., 2008). Our previous model showed how the combination of these neuromodulatory systems could produce effective action selection in robots (Krichmar, 2012).

The present model extends this prior work and takes into consideration the notion that the dopaminergic and serotonergic systems are in opposition. Specifically, the serotonergic system is inhibiting the dopaminergic system. One model that investigated these opponent interactions, suggested that tonic serotonin tracked the average reward rate and that tonic dopamine tracked the average punishment rate in a similar context, and speculated that a phasic serotonin signal might report an ongoing prediction error for future punishment (Daw et al., 2002). However, it has been difficult to find empirical evidence supporting these roles for tonic and phasic neuromodulation. Our prior modeling has shown that direct opponency between these systems is not necessary to achieve behavioral opponency (Asher et al., 2010, 2012; Zaldivar et al., 2010). In many cases there is an environmental tradeoff between the expected rewards and costs, and this can lead to opponency between active reward-seeking and withdrawn behavior. Indeed, by having different neuromodulatory systems handle different sensory events, this type of opponency emerged in the present model.

## Conclusions

The neurorobotic experiments presented here demonstrate that the opposition of the serotoninergic system with the dopaminergic system can lead to the type of anxious and curious behavior observed in animals. Whereas high levels of 5-HT led to withdrawn, anxious behavior by suppressing DA action, high levels of DA or low levels of 5-HT led to curious, exploratory behavior. Moreover, it was shown that top-down signals from the frontal cortex to these neuromodulatory areas were critical for handling both stressful and positive valence events. The action of the neuromodulatory system and its interaction with areas important for action selection and planning are in a fine balance. It was shown that if any of these systems become out of balance, due to lesions or changes to the efficiency of neuromodulatory signaling, aberrant behavior occurs. This may have implications for understanding mood disorders, obsessive-compulsive disorders, and anxiety.

### Conflict of interest statement

The author declares that the research was conducted in the absence of any commercial or financial relationships that could be construed as a potential conflict of interest.
